# Recurrent water deficit causes epigenetic and hormonal changes in citrus plants

**DOI:** 10.1038/s41598-017-14161-x

**Published:** 2017-10-20

**Authors:** Diana Matos Neves, Lucas Aragão da Hora Almeida, Dayse Drielly Souza Santana-Vieira, Luciano Freschi, Claudia Fortes Ferreira, Walter dos Santos Soares Filho, Marcio Gilberto Cardoso Costa, Fabienne Micheli, Maurício Antônio Coelho Filho, Abelmon da Silva Gesteira

**Affiliations:** 1 Departamento de Biologia, Centro de Genética e Biologia Molecular, Universidade Estadual de Santa Cruz, Ilhéus-Bahia, 45662-900 Brazil; 2 Departamento de Saúde, Faculdade de Ciências Empresariais, Santo Antônio de Jesus-Bahia, 44573-045 Brazil; 3 Departamento de Ciências Exatas e Tecnológicas, Universidade Estadual do Sudoeste da Bahia, Vitória da Conquista-Bahia, 45083-900 Brazil; 4 Departamento de Ciências Agrárias, Universidade Federal do Recôncavo da Bahia, Cruz das Almas-Bahia, 44380-000 Brazil; 5 Departamento de Botânica, Instituto de Biociências, Universidade de São Paulo, São Paulo, 05508-090 Brazil; 6 Embrapa-Mandioca e Fruticultura, Cruz das Almas-Bahia, 44380-000 Brazil; 7 CIRAD –UMR AGAP, F-34398 Montpellier, France

## Abstract

The present study evaluated the physiological, molecular and hormonal parameters from scion/rootstock interaction of citrus plants during recurrent water deficit. Responses of the Valencia (VO) scion variety grafted on two rootstocks with different soil water extraction capacities, Rangpur Lime (RL) and Sunki Maravilha (SM), during three successive periods of water deficit: plants exposed to a single episode of water deficit (WD1) and plants exposed to two (WD2) and three (WD3) recurrent periods of WD were compared. The combinations VO/RL and VO/SM presented polymorphic alterations of epigenetic marks and hormonal (*i*.*e*. abscisic acid, auxins and salicylicacid) profiles, which were particularly prominent when VO/SM plantswere exposed toWD3 treatment. Upon successive drought events, the VO/SM combination presented acclimatization characteristics that enable higher tolerance to water deficit by increasing transpiration (*E*), stomatal conductance (*g*_*s*_) and photosynthetic rate (*A*), which in turn may have facilitated the whole plant survival. Besides providing comprehensive data on the scion/rootstock interactions upon successive stress events, this study brings the first dataset suggesting that epigenetic alterations in citrus plants triggered by recurrent water deficit lead to improved drought tolerance in this crop species.

## Introduction

Restriction of water supply can severely limit plant growth, development and production^1,2^. Climate change is leading to global warming and more frequent and/or extreme drought events in many important agricultural regions of the world. Successive drought events have been shown to trigger permanent changes in plant responses in a sense that previous stress events can prepare the plant to overcome subsequent adverse conditions, thus characterizing a type of plant memory to these disturbances^3,4^. This stress memory can be developed at different life stagesandis associated with changes in plant physiological and molecular processes^2,5^.

Accumulating evidence indicates that epigenetic changes are behind the stress memory in plants^6,7^. Without any changes in the genome nucleotide sequences, stress events have been shown to permanently modulate gene expression via the activation or silencing via epigenetic mechanisms^3,8,9^. One of these epigenetic changes is the cytosine methylation in the DNA^7^, which is a reversible process depending on dedicated enzymes. Overall, the methylation in the coding or regulatory regions hinders the expression of target genes, whereas demethylation events are accompanied by the activation of genes in the eukaryotic genome^10,11^. A technique widely used in the evaluation of the methylation profile of the DNA is the Methyl-Sensitive Amplification of Polymorphism (MSAP). This technique uses the *Msp*I and *Hpa*II enzymes, which recognize 4-base sites (CCGG) and whose action depends on the methylation state of the internal cytosine residue^12^. Hence, MSAP allows simultaneously showing several regions of the genome giving rise to multi-location markers^6,7,13^. Alongside with other approaches, MSAP opens new opportunities for better understanding how DNA methylation is involved in stress-induced modifications in plant responses via epigenetic changes.

Over the years, increased attention has been dedicated to investigating the mechanisms underlying stress memory in crop species^2,14^. However, despite its agricultural importance, whether and how the memory to stress protects citrus plants during successive stress events remains to be explored. Water deficit has been shown to trigger a wide range of responses in citrus, including physiological, biochemical, hormonal and gene expression changes that improve the survival of these plants under water-limiting conditions^15,16^. Citrus rootstock combinations have been identified as a promising strategy to improve the fitness of this crop when challenged by moderate or severe drought^15,17,18^. Besides promoting several modifications in plantprimary and secondary metabolites, citrus rootstock combinations have also emerged as a particularly interesting approach for unraveling the plethora of physiological and molecular mechanisms behind the drought-induced responses in citrus plants.

Marked changes in citrus hormonal profile have been identified depending on the citrus rootstock combination and water availability in the soil^2,18^, particularly in the levels of abscisic acid (ABA), indole acetic acid (IAA) and salicylic acid (SA)^2,18^. As in many other plant species, drought promotes root and leaf ABA accumulation in citrus^17,18^, regulating stomatal opening and closure, and consequently adjusting water loss via transpiration according to the availability of this resource in the soil. Recent evidence indicates that ABA accumulation may be implicated in the stress memory in plants as the ABA levels in *Aptenia cordifolia* were higher in plants exposed twice to water deficit when compared to plants exposed to a single drought event^19^. Other plant hormones, such as auxins and SA, have also been implicated in mediating plant responses to water deficit^2,20–28^, including citrus^2,18^, however, whether these hormones are also implicated in the stress memory in plants remains to be elucidated.

Here, we have investigated the influence of recurrent water deficit on the physiological, molecular and hormonal changesinscion/rootstocks combinations containing rootstocks withdifferent soil water extraction capacities: Rangpur Lime (RL) and Sunki Maravilha (SM)^17,18,29^. After exposure to successive water deficit events, distinct polymorphic alterations of epigenetic marks depending on the scion/rootstocks combination were evaluated. Plants exposed to three recurrent water deficits exhibited the most pronounced physiological, molecular and hormonal alterations, which may reflect the acquisition of progressive tolerance to this environmental stress. This study brings the first dataset suggesting that epigenetic alterations in citrus plants triggered by recurrent water deficit lead to improved drought tolerance in this crop species.

## Results

### Morphological and physiological responses

**Figure 1:**
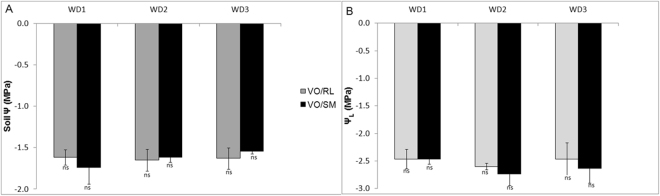
(**A**) Soil matrix potential (soil Ψ; MPa). (**B**) Leaf water potential before sunrise (Ψ_L_; MPa) under severe water deficit conditions for the different plant groups exposed to recurrent water deficit (WD1, WD2 and WD3), Valencia Orange/Rangpur lime (VO/RL-gray bar) and Valencia Orange/Sunki Maravilha (VO/SM-black bar) with Ψ ≥ 2,0 MPa. NS indicates no significance (P ≤ 0.05).

**Figure 2:**
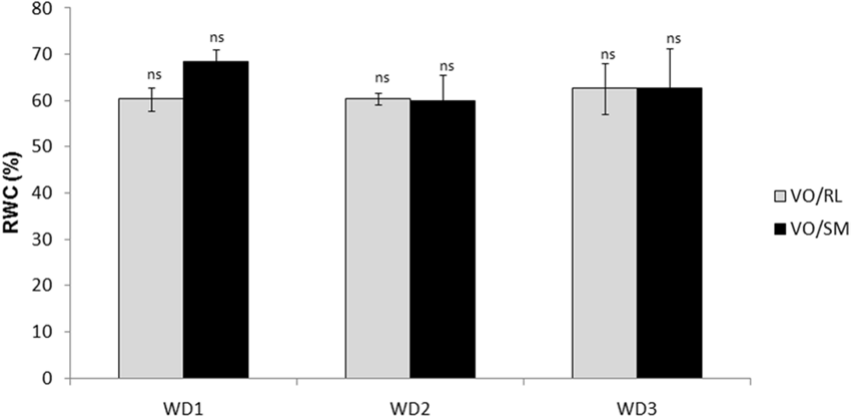
Relative leaf water content under severe water deficit conditions in the Valencia Orange/Rangpur lime (VO/RL-gray bar) and Valencia Orange/Sunki Maravilha (VO/SM-black bar) combinations in the 3rd stage of the recurrent water deficit treatment (WD1, WD2 and WD3). The values are averages (n = 3) and the bars indicate the standard error. NS indicates no statistical significance (P ≤ 0.05).

Similar total leaf area values were observed regardless ofthe genotype or treatment considered (Supplementary Fig. S1). Matric potential and leaf water potential below −1.5 MPa and −2.0 MPa, respectively, were observed at the end of the WD1, WD2 and WD3 treatments, which indicate the successful imposition of severe water deficit (Fig. 1A,B). RWC were also similar regardless of the scion/rootstock combination or treatment condition (Fig. 2). Upon rehydration, both scion/rootstock combinations reestablished their RWC and Ψ_L_ full capacity (Supplementary Fig. S2).

**Figure 3:**
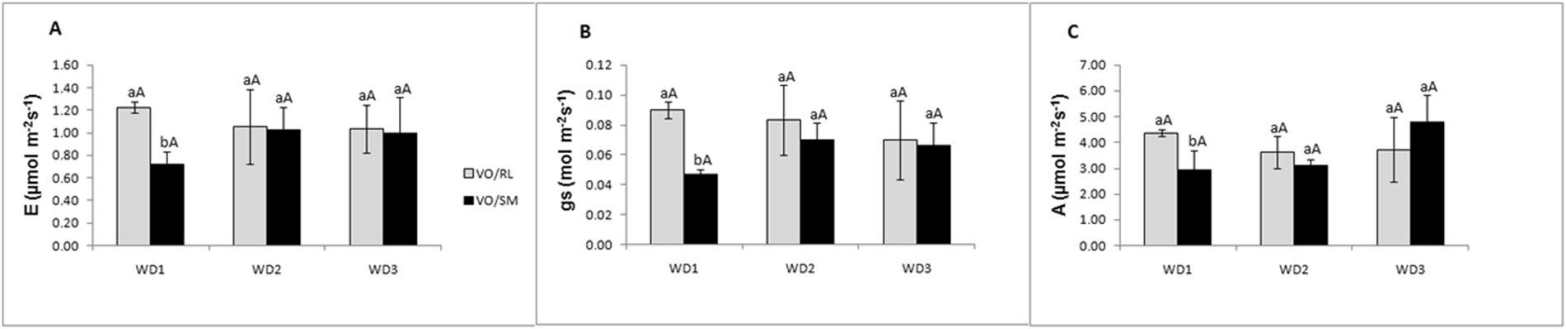
Transpiration (*E*), stomatal conductance (*g*_*s*_) and photosynthetic rate (*A*) in leaves of Valencia Orange/Rangpur lime (VO/RL-gray bar) and Valencia Orange/Sunki Maravilha (VO/SM-black bar) exposed to severe conditions of recurrent water deficit. Data represent the average ± SD of n = 3 individuals. Equal lowercase letters mean that there was no significant statistical difference between combinations. Uppercase letters indicateno significant statistical difference between the treatments of the combinations, Scott-Knott (p < 0.05).

Transpiration (*E*), stomatal conductance (*g*_*s*_) and photosynthetic rate (*A*) were affected by WD1 (Fig. 3A,B and C), being athigher values for VO/RL than in VO/SM plants. No statistical difference for WD2 and WD3 plants of the two scion/rootstock was observed, both within and between each period of severe water deficit, indicating a possible acclimatization of these plants after the first treatment of water deficit.

### MSAP analysis

The MSAP technique resulted in a total of 322 and 309 bands for the VO/RL and VO/SM combinations, respectively (Table 1). In the VO/RL combination, 229, 200, 201 and 195 total methylated bandswere found in plants exposed to control, WD1, WD2 and WD3 conditions, respectively; which indicates reduced methylation in drought-exposed plants. Conversely, in the VO/SM combination, 153, 192, 198 and 192 total methylated bandswere found in plants exposed to control, WD1, WD2 and WD3 conditions, respectively; which indicates increased methylation in drought-exposed plants.

**Table 1:** Different types of MSAP within the levels of methylated cytosine for Valencia Orange/Rangpur lime (VO/RL) and Valencia Orange/Sunki Maravilha (VO/SM) exposed to full irrigation (Control) and to the severe conditions of recurrent water deficit (WD1, WD2 and WD3).

MSAP band type^a^	VO/RL				VO/SM			
	CONTROL	WD1	WD2	WD3	CONTROL	WD1	WD2	WD3
I	93	122	121	127	156	117	111	117
II	97	29	41	28	27	30	52	52
III	70	45	49	52	38	71	56	52
IV	62	126	111	115	88	91	90	88
Total amplified bands	322	322	322	322	309	309	309	309
Total methylated bands^b^	229	200	201	195	153	192	198	192
Fully methylated bands^c^	132	171	160	167	126	162	146	140
MSAP%^d^	71.1	62.1	62.4	60.6	49.5	62.1	64.1	62.1
Hemi-methylated ratio(%)^e^	30.12	9.01	12.73	8.70	8.74	9.71	16.83	16.83
Fully methylated ratio (%)^f^	40.99	53.11	49.69	51.86	40.78	52.43	47.25	45.31

^a^Type I is the presence of bands in both EcoRI/HpaII and EcoRI/MspI enzymes and indicates the absence of methylation; type II are bands generated in EcoRI/HpaII enzymes but not in EcoRI/MspI enzymes; type III bands appear in EcoRI/MspI enzymes but not in EcoRI/HpaII enzymes; and type IV represents the absence of band in both enzyme combinations.

^b^Total methylated bands are II + III + IV bands.

^c^Fully methylated are III + IV bands.

^d^MSAP is a percentage ratio of total methylated bands (II + III + IV) to total amplified bands.

^e^Hemi-methylated ratio is a percentage ratio of total hemi-methylated bands (II) to total amplified bands.

^f^Fully methylated ratio is a percentage ratio of fully methylated bands (III + IV) to total amplified bands.

In the VO/SM combination, hemi-methylated ratio of 8.74%, 9.71%, 16.83% and 16.83% were found in plants exposed to control, WD1, WD2 and WD3 conditions, respectively; which indicates an increase in the rate of polymorphic bands particularly in WD2 and WD3 treatments. At first, this may indicate a methylation gain of the external cytosine for the treatments of recurrent water deficit, suggesting drought-induced hypermethylation. In contrast, in the VO/RL combination, hemi-methylated ratio of 30.12%,9.01%,12.73% and 8.70% were found in plants exposed to control, WD1, WD2 and WD3 conditions, respectively; which indicates a loss of methylation of the external cytosine in the control compared to recurrent water deficit treatments, thus suggesting drought-induced hypomethylation in this genotype.

**Figure 4:**
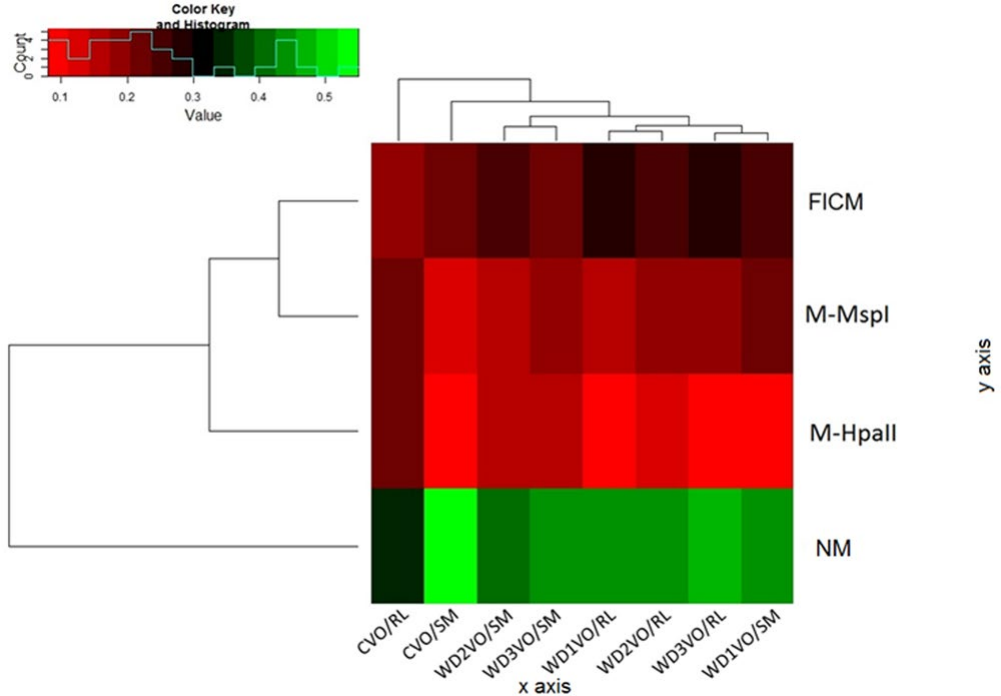
Heatmap representation ofthe methylation profile obtained with MSAP analysis digested with the MspI and HpaII enzymes to evaluate the VO/RL and VO/SM genotypes and the treatments (Control-C, and the different WD1, WD2 and WD3 deficit levels) in which NM characterizes the presence of bands cut with both enzymes and that there was non-methylation; M-HpaII presence of methylation from the cut with HpaII; M-MspI presence of methylation from the cut with MspI; and FICM identifies the group of bands in which there are no cuts where there are different types of full or internal methylation of methyl cytosine and/or mutation.

The heatmap representation of polymorphic patterns revealed a difference between the unmethylated (UM) profile for the methylated groups (M-HpaII, M-MspI and FICM) as highlighted by the color key, in which the unmethylated group is shown in green and the methylated group stands out in red (Fig. 4).

The frequency of methylation shown for the M-HpaII profile was similar in WD2 and WD3 for VO/SM, whereas it differed from WD1 with higher values for the methylation frequency for H-HpaII. This difference was also evident when comparing VO/SM WD2 and WD3 with VO/RL WD2 and WD3. Conversely, VO/SM WD presented higher frequency in the methylation profile in comparison to VO/RL WD3.

### Hormones

**Figure 5:**
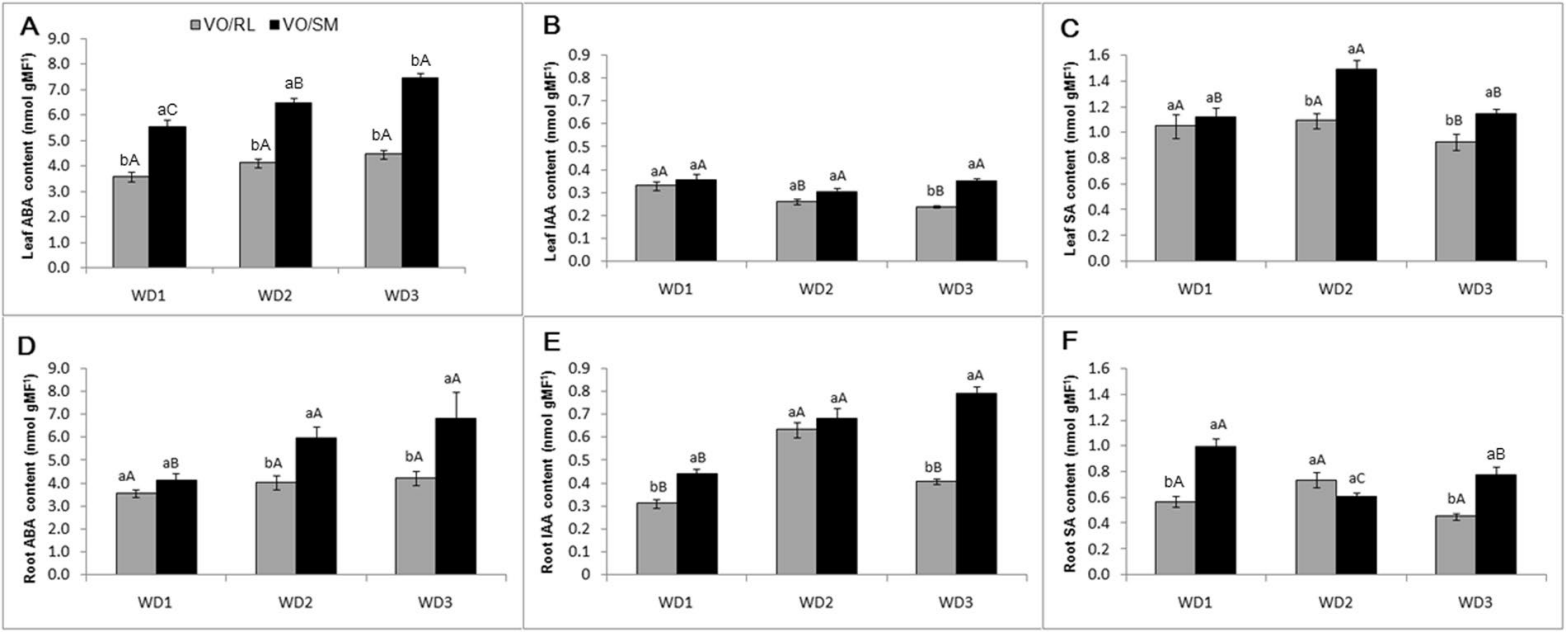
Endogenous ABA, IAA and SA levels in leaves (**A**,**B** and **C**) and roots (**D**,**E** and **F**) of Valencia Orange/Rangpur lime (VO/RL gray bar) and Valencia Orange/Sunki Maravilha (VO/SM-black bar) exposed to severe conditions of recurrent water deficit (WD1, WD2 and WD3). The values represent the averages (n = 5) ± the standard deviation. Capital letters compare differences between combinations. Lowercase letters compare differences within treatment. The Scott-Knott test (P > 0.05) was used for comparison of the averages.

Leaf ABA levels for the VO/SM combination showed significant differences between the three treatments (WD1, WD2 and WD3), with the highest values for the WD3 treatment (Fig. 5A). Plants exposed to more than one period of water deficit (WD2 and WD3) exhibited higher levels of ABA when compared to plants exposed to a single water deficit period (WD1). ABA content (Fig. 5D) was higher for the VO/SM combination across all treatments (WD1, WD2 and WD3) when compared to the VO/RL combination, with the exception of the levels of ABA in the roots for WD1. In the VO/RL combination, no significant differences in ABA levels were observed within the treatments of recurrent water deficit (WD1, WD2 and WD3), either in leaves or in roots.

In the VO/RL combination, IAA levels (Fig. 5B) were reduced in plants exposed to more than one recurrent water deficit event (WD2 and WD3). This was not observed in the VO/SM combination, which did not show significant differences between the three treatments (WD1, WD2 and WD3). In the VO/RL combination, root IAA levels (Fig. 5E) were significantly higher inWD1 and WD3 compared to the WD2. A progressive increase in root IAA was observed along the WD1, WD2 and WD3 treatment in the VO/SM combination.

In the VO/RL combination, WD1 resulted in higher leaf SA levels (Fig. 5C) compared to the WD2 and WD3 treatments. In contrast, in the VO/RL combination, the WD2 treatment resulted in higher leaf SA levels compared to WD1 and WD3. Furthermore, the highest root SA levels (Fig. 5F) in VO/SM were found in the WD1 treatment, while VO/RL did not present a significant difference.

### Activity of antioxidant enzymes

**Figure 6:**
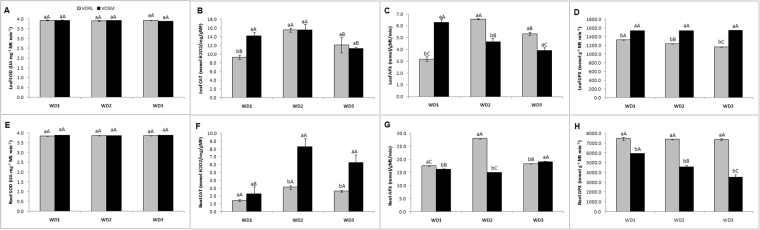
Superoxide dismutase (SOD), catalase (CAT), ascorbate peroxidase (APX), guaiacol peroxidase (GPX) activities in leaves (**A**,**B**,**C** and **D**) and roots (**E**,**F**,**G** and **H**) of Valencia Orange/Rangpur lime (VO/RL gray bar) and Valencia Orange/Sunki Maravilha (VO/SM-black bar) exposed to severe conditions of recurrent water deficit (WD1, WD2 and WD3). The values represent the averages (n = 3) ± the standard deviation. Capital letters compare differences between combinations. Lowercase letters compare differences within treatment. The Scott-Knott test (P > 0.05) was used for comparison of the averages.

Superoxide dismutase (SOD), catalase (CAT), ascorbate peroxidase (APX) and guaiacol peroxidase (GPX) activities were determined both in leaves and rootsalong the three recurrent drought events (Fig. 6). In both VO/RL and VO/SM combinations, leaf and root SOD activity remained unchanged all over the recurrent drought events and no significant differences (Fig. 6A). Leaf CAT activity in VO/SM plants did not show significant differences between the WD1 and WD2 treatments. However, both differed from WD3, with higher values for treatments WD1 and WD (Fig. 6B). In contrast, a marked increase in CAT levels in WD2 and WD3 compared to WD1 was observedin VO/SM but not in VO/RL root systems(Fig. 6F). Root CAT activity was significantly higher inVO/SM than in VO/RL plants for treatments WD2 and WD3.

In VO/SM plants, a progressive decrease in APX activity was observed along the three recurrent drought events (Fig. 6C). For the combination VO/RL, both leaf and root APX activitywere highest and lowest in WD2 and WD1 treatments, respectively. In VO/RL plants, leaf GPX remained unchanged along the three successive drought treatments, whereas the root activity of this enzyme was gradually reduced from the WD1 to the WD3 treatments (Fig. 6D,H). In contrast, recurrent drought had no significant influence on root GPX activityin VO/SM plants (Fig. 6H). Whereas leaf GPX values were always higher in VO/SM than in VO/RL plants, the opposite was observed in the root system.

## Discussion

Our previous results^18^ demonstrate that the differences in plant growth, photosynthesis, metabolism and hormonal balance between well-watered and droughted citrus plants are highly dependent on the scion/rootstock combination considered. Here, were compared plants of the same scion/rootstock combinations analyzed in Santana-Vieira *et al*.^18^ exposed to one, two or three successive cycles of drought to investigate the physiological and metabolic performance of these genotypes exposed to recurrent water limiting conditions. The comparison between droughted and well-watered plants of these same genotypes is presented in Santana-Vieira *et al*.^18^. Since the experimental design used in the present study allowed the plants in the WD1, WD2 and WD3 treatments to be evaluated at the same time and in the same conditions, thus avoiding physiological differences due to environmental changes, the WD1 was used as the control treatment.

Plant drought responses depend on the duration and severity level of the water deficitand vary within species and at different stages of plant development^30^. Pre-exposure to water stress may alter subsequent plant responses, producing faster and/or stronger reactions, which characterizes a form of plant stress memory^31^. This memory involves several mechanisms including the accumulation of intermediate compounds in the intercellular compartments, modification of key regulatory proteins, phosphorylation of MAPKs proteins and mainly epigenetic alterations that result in the silencing and/or activation of genes that alter the stress response when the plants are exposed to subsequent stress^3,32–34^.

Overall, maintenance of cell homeostasis avoiding the loss of water is among the first responses in plants, playing critical importance of minimizing the wilting is the loss of leaf turgor, yellowing and premature leaf senescence, and ultimately death^35^. Therefore, monitoring the relative water content (RWC), leaf water potential (Ψ_L_), stomatal conductance (*g*_*s*_), photosyntheticrate (*A*), transpiration (*E*) and growth can be a valuable tool to evaluate whether recurrent drought results in faster or stronger changes in plant physiological responses. A reduction in RWC leads to stomatal closure, and as the lower stomatal conductance also decreases photosynthesis and transpiration, the reduction in carbon assimilation limits plant growth^36,37^. Here, we demonstrate that both scion/rootstocks combinations exhibited similar drought-induced changes in water status (*i*.*e*. RWC and Ψ_L_) upon the application of one, two or three recurrent water deficits (Figs 1 and 2). However, VO/SM plants displayed a tendency to increase *E*, *g*_*s*_ and *A* values as the number of recurrent drought exposure increased, whereas no marked differences in photosynthesis and transpiration were observed in VO/RL plants subjected to a single or multiple drought events (Fig. 3). Besides possibly suggesting a gain of stress memory in VO/SM combination rather than in VO/RL plants, these findings are also in line with previous studies comparing VO/SM and VO/RL combinations, which have revealed that SM and RL rootstocks triggers distinct drought tolerance mechanisms when associated with the same scion^18^.

Although drought can be considered a multidimensional type of stress resulting in physiological, morphological, biochemical and molecular changes, the major drought-related plant responses are controlled at the molecular level, and certain signaling pathways play a central role in the coordination and communication of plant responses to water deficit^38^. One possible response of plants to stress is that they become more resistant to future exposure through the acquisition of memory that induces effects on plant development, leaving epigenetic marks in the genome; these tags will encode memories (*i*.*e*.proteins) for future responses as a result of stress^1^. MSAP data indicated that VO/SM and VO/RL plants exhibit significant differences in the frequency of total methylation upon recurrent drought exposure (Table 1). Although recurrent drought exposure promoted DNA methylation in VO/SM plants, the opposite was observed for the VO/RL combination. Hemi-methylated ratio also differed between VO/SM and VO/RL plants, indicating that drought promotes hypermethylation and hypomethylation in VO/SM and VO/RL combinations, respectively. Therefore, the distinct photosynthetic performance observed between both scion/rootstock combinations exposed to recurrent water deficit was accompanied by marked differences in the DNA methylation, which may explain the gain of memory to stress in VO/SM, but not in the VO/RL combination. Hypermethylation and hypomethylation of DNA are associated with repression and activation of transcripts, respectively^9,13^. As HpaII enzyme recognizes methylated cytosine externally, this external cytosine methylation characteristic may be related to the process of recurrent water deficit in citrus plants. Site-specific differential methylation pattern has also been observed in response to drought stress, *i*.*e*. methylation and de-methylation events taking place simultaneously, which indicates the involvement of drought stress-responsive epigenetic machinery targeting specific genes or regions of the genome^13,39^. During this process of survival and adaptation, the genomic cytosine methylation level and site-specific differential methylation changes due to different methylating and de-methylating enzyme activity in response to drought, leading to the activation and inactivation of the transcriptional process for specific genes related to drought tolerance^40–42^. In agreement with our findings, the MSAP technique also indicated marked changes in the frequency of DNA methylation in *Lolium perenne* upon water deficit exposure^6^.

Gene ontology analysis of genes associated with differentially methylated regions indrought-tolerant introgression rice lines under drought conditions revealed that the hypo- or hyper-methylated genes are mainly related to transport, stress responses, and transcription regulation^14^. According to these authors, several transcription factor genes were uniquely differentially methylated in drought-tolerant introgression rice lines under drought stress. In contrast, based on methylation-sensitive amplified polymorphism analysis, it has been shown that hypomethylation and hypermethylation are more frequent in drought-tolerant and drought-sensitive rice genotypes, respectively, under drought conditions^13^. DNA hypermethylation was also induced by drought in peas^43^. Moreover, extreme temperatures, such as heat and cold stress, affect the expression of genes involved due to DNA methylation alterations DNA methylation has also been intimately associated with the cold- and heat-stress-induced gene expression^44,45^.

Hormonal changes may also be associated with the gain of memory to stress^19,46^ and a strong link between DNA methylation and plant hormone signaling has also been described during various physiological processes^47–49^. Among plant hormones, ABA regulates many physiological and biochemical processes of acclimatization and/or tolerance, some of them common to different stress conditions^50^. Stressful environmental conditions, particularly drought, generally promote ABA biosynthesis and accumulation^51^ and this hormone plays a major role in regulating the water status by controlling stomatal aperture and promoting enzymes and other proteins involved in cell dehydration tolerance, among other physiological and biochemical responses^52^. ABA may act asa long-distance water stress signaling molecule as drought-exposed roots synthesize and transport ABA to the xylem where it will be translocated to the canopy and regulate stomatal opening and growth of the aerial parts^53–55^. Previous findings revealed that SM and RL genotypes display marked differences in hormonal response when challenged by water deficit^18^. Overall, canopies grafted onto SM exhibit a tendency to increase leaf ABA levels when compared to those grafted onto RL rootstocks^18^.

During the first cycle of water deficit (WD1), VO/RL and VO/SM photosynthetic rates were significantly different. Considering WD1 as a reference, this difference was not observed in WD2 and WD3 due to a slight decrease and increased in the photosynthetic rate of VO/RL andVO/SM combinations, respectively. This data agrees with the increase in ABA content in leaves and roots of the VO/SM combination, which was not observed in the VO/RL combination. This suggest an important influence of the root system on the acclimation capacity of citrus against recurrent drought events. In line with this, our previous findings^18^ indicate that SM rootstocks facilitates plant survival by promoting drought tolerance by increasing ABA, which in turn triggers water-saving responses^31,56,57^.

Working with *Arabidopsis* ^56^, confirms that guard cells exhibit transcriptional memory similar as to what occurs to some genes and that elevated concentrations and/or increase of ABA sensitivity have been a strong memory mechanism in guard cells which stretches throughout many periods of drought and rehydration. Also^19^, evaluating re-occurrence of water deficit in *Aptenia cordifolia* plants, analyzed carbon assimilation (*A*) and stomatal conductance (*g*_*s*_), reporting no variation between plants of the second (SS) in comparison to those from the first (CS) stress cycle. However, as to ABA content, significant differences between the plant groups were noticed, whereas the SS plants presented higher values in comparison to the CS plants. The authors concluded that the *Aptenia cordifolia* plants had a memory capacity in the SS plants and that the chances of the ABA levels in those plants were probably modulating the changes in the redox state of the cell, such as signaling for less oxidative damage.

Increase in ABA levels in plants undergoing recurrent water deficit suggests an influence of this plant hormone on growth regulation, osmotic adjustment and antioxidant responses^19,46,58^. Also, previous studies have shown that ABA plays a protective role under recurrent drought via gene expression reprogramming^19,31,56^. Considering WD1 asreference, our data indicate that recurrent water deficit significantly promotes ABA levels in VO/SM plants, whereas no marked changes are observed in individuals of the VO/RL combination (Fig. 5A,B). Therefore, this contrasting hormonal response observed between VO/SM and VO/RL plants may alsobe associated with marked changes in drought-induced DNA methylation pattern detected between these genotypes. Recent discoveries reveal that besidesgenetic regulation, epigenetic regulation plays a key role inABA-mediated plant processes^56,59^. ABA might mediate stomatal response by chromatin remodeling through changes in histone acetylation status at certain loci. In tomato,the linker histone H1 variant, H1-S, was induced by drought through an ABA-dependent pathway^59^.

Besides ABA, previous findings have also revealed that drought-induced impact on auxin and SA are markedly different betweenVO/SM and VO/RL plants^18^. In the present study, a clear correlation between root ABA and IAA levels was observed in VO/SM plants, *i*.*e*. a rise in both hormones was observed as the number of recurrent drought events increased. It is possible that the increase in ABA levels favored by the VO/SM combination promoted leaf IAA levels, thus facilitating growth rate due to regulation of transpiration and carbon assimilation^60–62^. The impact of epigenetic alterations in auxin synthesis, transport and signal transduction has been recently described^49,63^, although the mechanisms behind the influence of DNA methylation on auxin metabolism and signaling still remains far from elucidated. Moreover, recurrent water deficit progressively decreased root SA levels in VO/SM plants (Fig. 5), which agrees with the reports indicating that water deficit triggers changes in SA^64–66^ and this hormone plays an important role in stomatal closure, thus favoring drought tolerance^67^.

Drought tolerance also requires the presence of a highly efficient antioxidant metabolism to cope with the stress-derived increased in reactive oxygen species (ROS) production. In high concentrations, activated or partially reduced oxygen species such as hydrogen peroxide (H_2_O_2_) and the superoxide radical (O_2_^•−^), can modify different biomolecules, such as oligonucleotides, sugars, proteins and lipids, leading to the oxidative destruction of the cell^68,69^. Key components of the plant cell antioxidant system are the so-called antioxidant enzymes, such as SOD, CAT, APX and GPX and the non-enzymatic system, including the carotenoids, alkaloids, ascorbate and glutathione^70–72^. Under drought, SOD is the first line of plant defense against ROS as is activity convertsO_2_^•_^into H_2_O_2_. Since SOD removes O_2_^•_^, this enzyme plays an important role under moderate rather than severe water deficit conditions^73–76^. In line with this, no marked changes in SOD were observed when VO/RL and VO/SM plants were exposed to recurrent severe drought events (Fig. 6).

Catalase, APX and GPX are the main enzymes responsible for H_2_O_2_ elimination in plants; therefore, their coordinated action is key to maintain the levels of this reactive species under strict control. The higher rootCAT levels in WD2 and WD3 in VO/SM plants, suggest that this enzyme may play a key role in conferring increased defense against drought-derived ROS in this particular scion/rootstock combination. Interestingly, VO/SM but not VO/RLplants exhibited progressive changes in root CAT and GPX and in leaf APX, which further indicates that the VO/SM combination confers particular stress memory mechanisms leading to increased the defenses against excessive ROS production,and consequently more efficient metabolic traits to cope with severe and recurrent drought conditions.

Therefore, several lines of evidence indicate that recurrent drought triggered memory to stress more clearly in VO plants grafted onto SM than in VO plants grafted onto RL. Firstly, only VO/SM plants exhibited significant increments in photosynthetic parameters as the number of drought exposures increased. Secondly, progressive changes in hormonal balance over the multiple water deficit events were also only observed in VO/SM plants. Thirdly, progressive changes in key components of the antioxidant machinery along the recurrent drought events were only observed in VO/SM plants, thus promoting the defenses of these plants against excessive ROS production. Forth,frequencies of methylated polymorphic fragments identified by the MSAP technique were markedly different between VO/RL and VO/SM plants. As the MSAP data were obtained in leaf samples, thedirect influence of the rootstocks on the frequencies of methylated fragments is of particular notice. Moreover, as epigenetic changes in plantscan be transmitted by meiosis and mitosis, the genetic and physiological changes observed in VO/SM plants exposed to recurrent drought could be maintained during the subsequent development of these organisms. Thus, we can suggest that the transfer of stress memory over time may allow the use of buds of these drought-treated individuals in new grafting events as an alternative in citrus management.

Altogether, our findings indicate that epigenetic alterations involving DNA methylation are involved in drought tolerance in citrus, and hormonal changes triggered by recurrent drought may facilitate preparing the plants for future exposure to water-limiting conditions.

## Methods

### Plant material and drought treatment

‘*Rangpur Lime*’ (RL) and ‘*Sunki Maravilha*’ mandarin (SM) citrus varieties, known to exhibit different drought response patterns^17,18,29^, were used as rootstocks to scions of the *Citrus sinensis (L*.*)* Osb.‘*Valencia’* orange (VO) variety. After grafting, the plants were transferred to 45-L pots containing Plantmax®, washed sand and clay (2:1:1). The plants were kept in an anti-aphid screen at Embrapa Cassava and Fruit Crops (Embrapa Mandioca e Fruticultura), with daily irrigation. The climaticconditions during the drought stress are presented in Supplementary Fig. S3. NPK and micronutrient fertilizers were applied every two weeks until plants reached 2 years of age. After this period, plants of each scion/rootstock combination (VO/RL and VO/SM) were exposed to up to three periods of water deficitshown in Supplementary Fig. S4. Between each water deficit event, plants were maintained underfield capacity, periodically pruned and fertilized.

The recurrent water deficit experiment was installed a completely randomized design, and included three experimental groups as shown in Supplementary Fig. S4. The first stage began in October 2014, when the plants were submitted to two water regimes: i) Group 1 included plants maintained under field capacity, referred to as control condition (C1); and ii) Groups 2 and 3 included plants exposed to the water deficit treatment (D1). The second stage began in March 2015, with the application of two water regime conditions: i) plants of Group 1 remained under field capacity, referred to as control plants (C2) from then on; ii) plants of Group 2, which were treated with water deficit during stage one, were kept under field capacity (R1); and (iii) plants of Group 3 were exposed to the treatment of water deficit for the second time (D2). During the third and last stage, which began in May 2015, all plants were exposed to water restriction as follows: Group 1 (WD1) represents the plants exposed to the water deficit treatment only in stage three; Group 2 (WD2) represents plants exposed to the water deficit treatment in stages one and three, i.e. double exposure with interval; and Group 3 (WD3) represents the plants exposed to the water deficit treatment in all three stages, thus corresponding to the recurrent water restriction conditions. In the present study, the data presented refer to the third stage of this experiment. Drought was developed with gradual loss of soil water content. Soil moisture was monitored daily, using a time domain refractometry (TDR) probe. When the water potential in the leaves of the plants reached values below −2.0 MPa, leaf and root samples were harvestedand the plants were then rehydrated.

### Leaf area, leaf relative Water Content (RWC), leaf water potential (Ψ_L_) and matrix potential (soil Ψ)

Total leaf area was determined as described by Neves *et al*. (2013)^17^ before the last drought stress event. Leaf relative water content in the leaves was determined on the day of harvesting according to Barrs and Weatherley (1962)^77^. Leaf water potential (Ψ_L_) was determined before dawn, using a Scholander pressure chamber (M670, PMS Instrument Co., Albany, OR, USA). Ψ_L_ was measured every two days after confirming that the photosynthetic parameters obtained by an infrared gas analyzer (IRGA) were in fact decreasing. Leaves were detached from the plants using a razor, and they were immediately used for the Ψ_L_ measurements. The leaves and roots of the plants under severe drought were harvested when they reached Ψ_L_ ≤ −2.0 MPa (drought samples) or 48 h after the rehydration (rehydration samples). The soil matric potential (soil Ψ) was monitored on a daily basis, according to the soil moisture observed by the TDR probe, and associated with the potential matric values obtained with the psychrometer model WP4 Dew point potential Meter.

### Photosynthetic parameters

The net rate of photosynthesis (*A*), stomatal conductance (*g*_*s*_) and transpiration (*E*),were measured every two days in fully expanded mature leaves, which were selected and marked before the beginning of the experiment. The gas exchange measurements (*A*, *g*_*s*_ and *E*) were carried out using the portable LCpro-SD IRGA (ADC biotech-scientific Limited, UK) with photosynthetically active radiation (PAR) from 1000 μmol photons flux density m^−2^ s^−1^, while leaf temperature, air humidity and CO_2_ concentration were determined by the environment. Once readings had stabilized, measurements between the 8 and 11 a.m. on one leaf of each plant, were performed.

### DNA extraction

Total genomic DNA was extracted using the CTAB method^78^ and the extracted DNA was quantified by the Qubit Fluorometer 2.0 Invitrogen.

### Genotyping Protocol by MSAP

The MASP-type markers were obtained according to Tang *et al*.^6^, using the same protocol as for the AFLP-type markers.

### Hormonal measurements

The endogenous levels forindole acetic acid (IAA), salicylic acid (SA) and abscisic acid (ABA) were determined via gas chromatography-tandem mass spectrometry-selecting ion monitoring (GC-MS-SIM)as described in Santana-Vieira *et al*.^18^.

### Activity of antioxidant enzymes

Briefly, samples were ground in liquid nitrogen in the presence of 0.7% (w/w)polyvinylpyrrolidone (PVP) and subsequently homogenized in the appropriate extraction buffer^16^. After sonication (8 pulses of 5 s each, with intervals of 10 s) and centrifugation (13,000 *g*, 10 min, 4 °C), the supernatant was used to determine the SOD, CAT, GPX and APX activities as described in Gonçalves *et al*.^16^.

### Statistical analysis

The experiment was carried out in an entirely randomized design (ERD), including 3 replicates for each group of water deficit treatments (WD1, WD2 and WD3). The analysis of variance (ANOVA) and the Scott-Knott’s test were calculated using the Sisvar software to detect differences between combinations of the same comparison group, between stress situations within the same combination, as well as the interaction between combinations and treatments, with P value < 0.05. Three replicates (n = 3) were used to analyze soil and leaf water potential, as well as to determine leaf area, relative water content (RWC) and photosynthetic parameters (*A*, *gs* and *E*); and five replicates (n = 5) were used to determine hormone contents. Pools were used for the treatmentsand the frequency test was evaluated using the Heatmap package^79^ implemented by the R software (Core Development Team, 2016) and the gplots and R Color Brewer packages for the methylation analyzes by the MSAP technique.

## Electronic supplementary material

Supplementary Information
