# Supplementary material for: Recurrent water deficit causes epigenetic and hormonal changes in citrus plants
Source: Sci Rep. 2017 Oct 20;7:13684. doi: 10.1038/s41598-017-14161-x (PMC5651809; doi:10.1038/s41598-017-14161-x)
Supplement: Supplementary file 1 — Supplementary Information [file 41598_2017_14161_MOESM1_ESM.doc]

**Supporting file**

**Title**

**Recurrent water deficit can cause epigenetic and hormonal changes in citrus plants**

**Authors**

Diana Matos Neves^1^ Lucas Aragão da Hora Almeida^2^ Dayse Drielly Souza Santana-Vieira^3^ Luciano Freschi^4^ Claudia Fortes Ferreira^5^ Walter dos Santos Soares Filho^5^ Marcio Gilberto Cardoso Costa^1^ Fabienne Micheli^1,6^ Maurício Antônio Coelho Filho^5^ Abelmon da Silva Gesteira*^1,5^

^1^ – Departamento de Biologia, Centro de Genética and Biologia Molecular, Universidade Estadual de Santa Cruz, Ilhéus – Bahia, 45662-900, Brazil

^2^ – Departamento de Saúde, Faculdade de Ciências Empresariais, Santo Antônio de Jesus – Bahia, 44573-045, Brazil

^3^ – Departamento de Ciências Exatas and Tecnológicas, Universidade Estadual do Sudoeste da Bahia, Vitória da Conquista - Bahia, 45083-900, Brazil

^4^ – Departamento de Botânica, Instituto de Biociências, Universidade de São Paulo, São Paulo 05508-090, Brazil

^3^ – Departamento de Ciências Agrárias, Universidade Federal do Recôncavo da Bahia, Cruz das Almas - Bahia, 44380-000, Brazil

^5^ – Embrapa – Mandioca e Fruticultura, Cruz das Almas - Bahia, 44380-000, Brazil

^6^ – CIRAD –UMR AGAP, F-34398 Montpellier, France

**Figure Captions**

**Fig. S1:** **Leaf area water deficit treatment.** Leaf area for the combinations of Valencia Orange/Rangpur lime (VO/RL-gray bar) and Valencia Orange/Sunki Maravilha (VO/SM - black bar) measured at the beginning of the 3rd stage of the water deficit treatment. NS indicates no statistical significance (P≤0.05).

Fig. S2: Relative water content in the leaves and Leaf water otential at dawn (Ψ_L_; MPa) during severe and rehydrated condition in plants submitted to recurrent water deficit. A: Relative water content in the leaves and B: Leaf water potential at dawn (Ψ_L_; MPa) during severe and rehydrated condition in plants submitted to recurrent water deficit ((WD1, WD2 e WD3),Valencia orange/Rangpur Lime (VO/RL – medium gray bar represents severe water deficit and dark gray bar represents rehydrated plants); and Valencia orange/Sunki Maravilha (VO/SM – black bar represents severe water deficit and dark gray bar, rehydrated). Data represents the average ±EP for n=3 plants. Same small letters indicate no significant statistical difference between treatments for each combination while same capital letters indicates no significant statistical difference between combinations and NS indicates no statistical significance for the Scott-Knott test ( p< 0.05).

Fig. S3: Monitoring of climatic conditions: Microclimate conditions under the screen where the experiment was performed. The daily maximum and minimum temperatures (°C) and air relative humidity (%) for the days of the experiment are represented as lines and bars, respectively.

Fig. S4: Experimental design of the recurrent water déficit. For the first (1^st^) water stress (C1 = control plants from the first imposition of water defict, D1 = plants under water deficit for the first time); second (2^nd^) stress (C2 = control plants for the second stress, R1 = plants maintained at full water capacity and D2 = plants under water déficit for the second time); third (3^rd^)stress (WD1 = plants under water deficit for the first time, WD2 = plants under water deficit for the second time with an interval and WD3 = plants under water deficit for the third time.


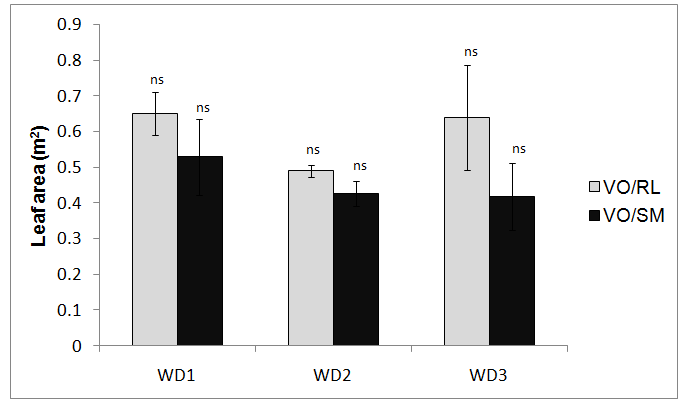


Fig. S1


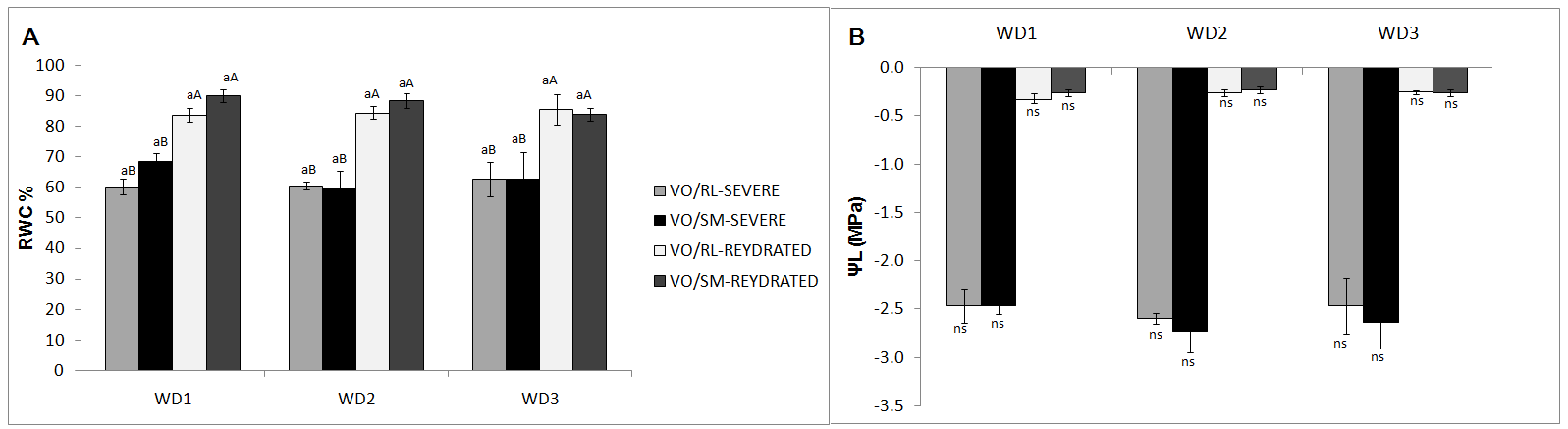
**Fig. S2**


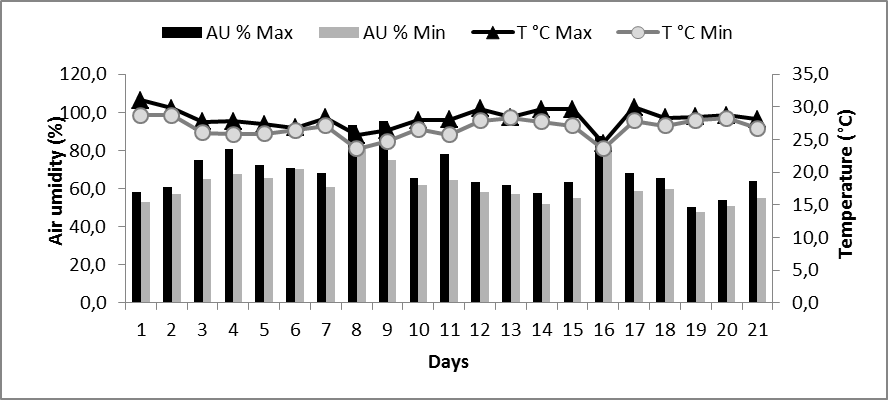


**Fig. S3**


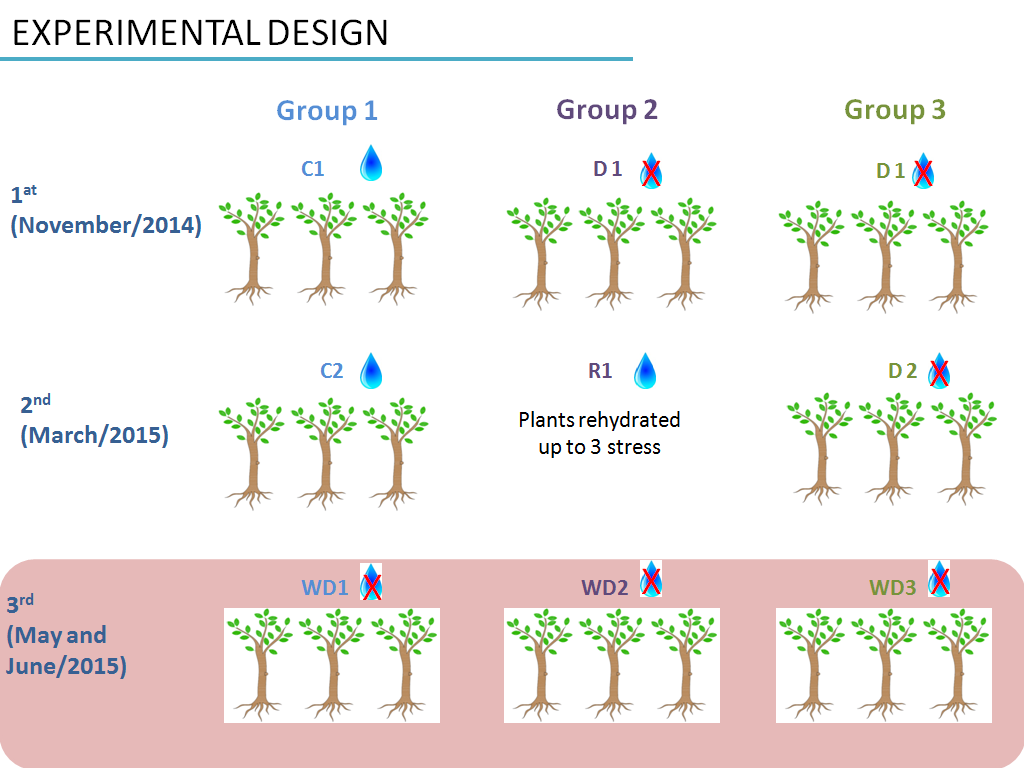


Fig. S4
